# Oral Rehabilitation of Hypodontia Patients Using an Endosseous Dental Implant: Functional and Aesthetic Results

**DOI:** 10.3390/jcm8101687

**Published:** 2019-10-15

**Authors:** Sameh Attia, Heidrun Schaaf, Thaqif El Khassawna, Deeksha Malhan, Katharina Mausbach, Hans-Peter Howaldt, Philipp Streckbein

**Affiliations:** 1 Department of Cranio Maxillofacial Surgery, Justus-Liebig University Giessen, 35392 Giessen, Germany; heidrun.schaaf@uniklinikum-giessen.de (H.S.); HP.Howaldt@uniklinikum-giessen.de (H.-P.H.); Philipp.Streckbein@uniklinikum-giessen.de (P.S.); 2 Experimental Trauma Surgery, Faculty of Medicine, Justus-Liebig University of Giessen, 35392 Giessen, Germany; Thaqif.ElKhassawna@chiru.med.uni-giessen.de (T.E.K.); Deeksha.Malhan@chiru.med.uni-giessen.de (D.M.); 3 Department of Prosthodontics, Justus-Liebig-University Giessen, 35392 Giessen, Germany; Katharina.A.Mausbach@dentist.med.uni-giessen.de

**Keywords:** tooth agenesis, hypodontia, dental implants, quality of life, implant success

## Abstract

Hypodontia often leads to limited bone availability of the alveolar ridges. Oral rehabilitation of severe hypodontia patients is challenging. In this retrospective study, we evaluated the functional and aesthetic results after dental implants in hypodontia patients, corroborated by Albrektsson implant success criteria. Over a period of 15 years (2000–2015), a total of 43 patients were diagnosed with hypodontia and 165 dental implants were inserted. Six patients who received 10 implants were lost in the follow-up. We examined 155 implants in 37 patients between December 2015 and May 2017. Besides family history, patients evaluated the general satisfaction, functionality, and aesthetics of the implants. Study subjects were between 17 and 44 years old (mean ± SD: 21.4 ± 5.6). Hypodontia patients were missing one to five teeth (*n* = 28), whereas patients diagnosed with oligodontia (≥6 missing teeth, *n* = 9). In this study, 24 patients (64.9%) with hypodontia had a positive family history; the remaining 13 patients had no family member with hypodontia. The final follow-up time ranged between 5 and 189 months after implant placement. Orthodontic treatment was performed in 32 patients (86%) before implant placement. Rehabilitation resulted in 62% of the cases being treated with 1–2 implants and 38% treated with 3–15 implants. However, out of 155 inserted dental implants, 18 implants failed to meet Albrektsson criteria, under which two implants were removed. Only autografts were used for bone augmentation with 97 implants. More than two-thirds of the patients showed high general satisfaction and masticatory function (69.4%) as well as phonetic ability (80.6%). The aesthetic outcome was rated as excellent by 17 patients (47.2%). The findings emphasize the importance of interdisciplinary treatment of hypodontia, leading to a satisfactory, functional, and long-term fixed prosthodontics using dental implants.

## 1. Introduction

Hypodontia is the most common congenital anomaly in tooth development [1,2,3] that results in tooth agenesis. A cut-off of five missing teeth differentiates between hypodontia and severe hypodontia (oligodontia) [4]. Studies reported a 2–10% incidence rate of hypodontia in the secondary dentition, with women being more affected than men [5,6,7]. Although tooth agenesis be associated with other syndromes or occur as a result of genetic factors [8,9,10,11,12], the exact mechanism of hypodontia is not fully understood [13].

Hypodontia may result in complex sociopsychological problems, especially during puberty and in the case of face disproportion. Therefore, treatment options with good long-term functional and aesthetic rehabilitation results are urgently needed [1]. However, a multidisciplinary team is often required with different competences, including maxillofacial surgeons, orthodontists, prosthodontists, speech pathologists, and psychologists, to treat severe hypodontia [14,15,16,17,18].

Patients with face disproportion and with either class II or III malocclusion need a combination of orthodontics and orthognathic treatment before teeth replacement [14,16]. Conservative prosthetic treatment options have limitations in severe hypodontia cases and might lead to unsatisfactory results [15,19]. Besides patient dissatisfaction, removable partial dentures have a short life span of 3.5–4 years due to wear and fracture [17,20]. In contrast, replacing missing teeth with dental implants in hypodontia and other indications has been shown to achieve adequate success in terms of function, aesthetics, dental rehabilitation, and long-term survival [5,18,21,22,23]. 

Adequate alveolar bone and keratinized gingiva have been implicated in successful implant insertion [24]. The presence of remaining deciduous teeth preserves the buccal bone, and their early loss leads to alveolar bone atrophy [10]. However, the surgical removal of an ankylosed deciduous tooth is often associated with local bone loss. Therefore, augmentation before dental implant placement is often required in such situations [25]. Iliac crest bone graft is the gold standard for jaw augmentation and is mainly used in patients with large amounts of alveolar bone atrophy [26]. Alternatively, intraoral bone harvesting from the retromolar area, chin, or maxilla can be performed to rebuild the atrophic area [27,28]. Autografts provide important properties for bone formation, such as osteogenesis, osteoconduction, and osteoinduction [26]. Besides autografts, an allograft, xenograft, or a combination of grafts have been successfully implemented [29]. 

Long-term aesthetic, functional, and satisfaction results in hypodontia patients are not adequately addressed in the literature [23]. The survival of dental implants is not an indication of their success [30]. Therefore, a comparison between implant survival and patient satisfaction as a success criterion has, to the best of our knowledge, not yet been addressed. The new generations of implants are designed with improved material, geometry, and concepts in comparison with the early models.

Taken together, these reasons inspired us to revisit the decades-old standards of implant success criteria by including not only survival but also functionality and aesthetics as defined by patients. Thus, clinically qualified personnel can improve approaches to meet the needs of patients within the limits of successful treatment prerequisites. Therefore, we reassessed the same subjects reported in a recent study addressing implant survival and success in patients [30] to evaluate aesthetic and functional treatment results. In this retrospective study, we compared these results with implant success according to the Albrektsson criteria.

## 2. Materials and Methods

### 2.1. Ethics and Privacy

This study was approved by the ethics committee of the Faculty of Medicine at Justus-Liebig University (Giessen, Germany; approval no. 209/15). The anonymity of the patients was ensured by assigning each patient a number; the statistical evaluation then occurred only with the assigned numbers. 

### 2.2. Study Design

This retrospective, clinical, observational study included all hypodontia patients *(n* = 43) treated with dental implants in the period from January 2000 to December 2016 at the Department of Oral and Maxillofacial Surgery, University Hospital Giessen, Germany.

### 2.3. Exclusion Criteria

The following exclusion criteria were considered in this study: (1) patients physically unable to respond to the questionnaire; (2) patients with a disease or take a medicine that direct influences osseointegration; and (3) female patients who were pregnant at the time of the follow-up.

### 2.4. Surgical Procedures

Recommended and standardized surgical protocols were followed for oral implants. The dental implant site and size were based on preparatory diagnostic aids and clinical assessment including bone quality. Drilling was maintained below 800 rpm under constant irrigation with normal saline. Implant placement was attempted using an electrical micro motor with a maximum torque of 50 N·cm.

### 2.5. Measured Variables 

We interviewed 37 (21 women and 16 men) of the 43 (25 women and 18 men) patients using a customized questionnaire: 28 with hypodontia and nine with severe hypodontia. The questionnaire was used to collect data regarding the patients’ sex, general diseases, family history related to hypodontia, smoking behavior, general satisfaction with dental implants, chewing ability, pronunciation, and aesthetic outcome.

General satisfaction, chewing ability, pronunciation, and aesthetic outcomes were assessed using the German grade ranking: grade 1 = excellent, grade 2 = good, grade 3 = acceptable, grade 4 = adequate, grade 5 = poor, and grade 6 = unsatisfactory. Types of prosthetic restorations and their complications (e.g., crown and abutment loosening, screw fracture, and ceramic chipping) were recorded. 

Photographic documentation was recorded for all cases at the follow-up examination. This included a frontal image as well a picture of the upper and lower jaw. If removable dentures were present, a photo was captured with and without dentures. Implant loss was defined as the complete removal of an implant.

Implant success was evaluated using the Albrektsson implant success criteria [31], as reported in a previous study [30]. In the present study, we analyzed the Albrektsson criteria for successful and failed dental implants with regard to: augmentation, augmentation type, prosthetic type, implant company, general satisfaction, aesthetics, speech, and chewing function.

### 2.6. Statistical Analysis

Statistical analysis for this study was conducted using the statistical package PASW 24.0 (IBM Corporation, Armonk, NY, USA). Data were examined for their Gaussian distribution and were found to be non-parametric. Frequency analysis was conducted using the chi-square test to compare the implant success and failure. Data are presented as bar graphs with whiskers of the standard error of means.

## 3. Results

A total of 43 patients (25 women and 18 men) were diagnosed with hypodontia and treated with 165 dental implants. Six patients did not respond to follow-up. Therefore, 37 patients (21 women and 16 men) were included in this analysis. Twenty-eight patients were diagnosed with hypodontia when missing one to five teeth, whereas the nine patients diagnosed with severe hypodontia were missing 6–20 teeth.

The final follow-up time was recorded per implant (not patients). The follow-up ranged between a minimum of 5 and maximum of 189 months, with a mean ± SD of 109.92 ± 54 and a median of 123 months.

**Figure 1:**
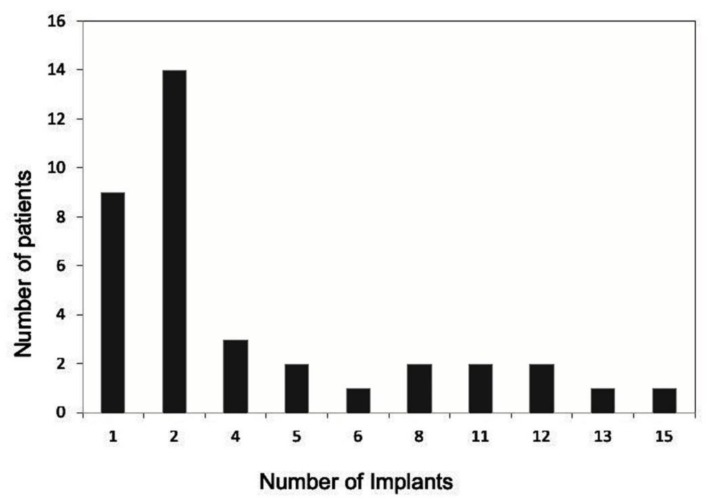
Number of inserted implants per patient (*n* = 155).

A total of 155 dental implants were inserted in hypodontic areas. Two implants were rated as failures and were removed (1.3%); this resulted in a survival rate of 98.7% (Table 1). The implant success according to Albrektsson criteria was 88.4% with 18 implant failures [30]. Figure 1 shows the number of inserted implants in each patient. The medical conditions of patients were recorded prior to treatment (Table 2). Inserted dental implants in relation to missing teeth were categorized as follows: 50 incisors, 21 canines, 68 premolars, and 16 molars (Supplementary Table S1).

**Figure 2:**
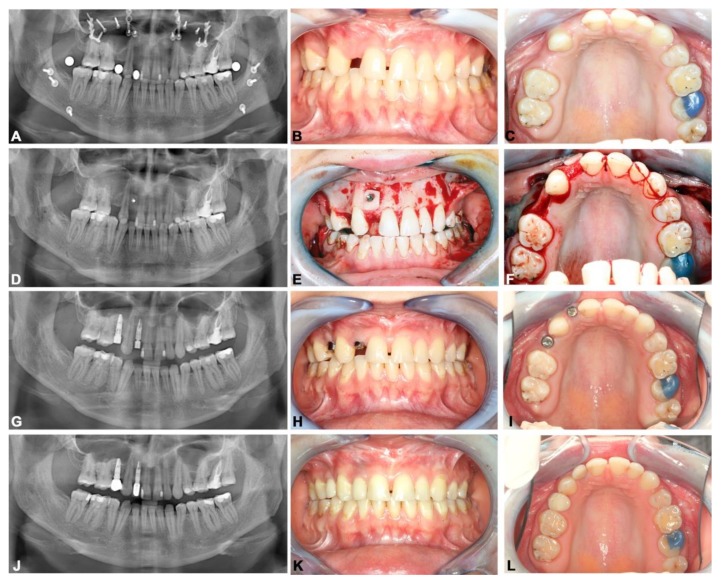
Dental rehabilitation of patient with hypodontia. (**A**–**C**) Panoramic radiograph and intraoral photos showing the situation after orthognathic surgery and teeth gaps in the left maxillary: first premolar and lateral incisor region. (**D**–**F**) Panoramic radiograph and intra-operative photos showing bone augmentation in the left maxillary lateral incisor region. (**G**–**I**) Panoramic radiograph and intraoral photos after the exposure of the implants. (**J**–**L**) Panoramic radiograph and intraoral photos after 54 months of prosthetic rehabilitation.

The following representative case reflects the diagnostic and treatment procedure and outcome. A 23-year-old female patient was referred to our department with hypodontia and gaps in the left maxillary: first premolar and lateral incisor regions. The patient had class III malocclusion and was orthodontically and orthognathically treated. Clinical and radiographical investigation showed bone atrophy in the maxillary lateral incisor region, meaning that we augmented this area with bone harvested from the retromolar region. Three months later, we inserted two Xive Plus^®^ dental implants (Dentsply Friadent, Mannheim, Germany). In February 2013, the implants were exposed and two single ceramic crowns were inserted. A follow-up investigation, including the assessment of functional and aesthetic outcomes, was performed 56 months later (Figure 2).

The general condition of patients, as well as existing allergies at the time of follow-up, were documented. The smoking behavior of the patients was also noted. Table 2 lists the patients with general conditions, allergies, and regular nicotine consumption.

**Figure 3:**
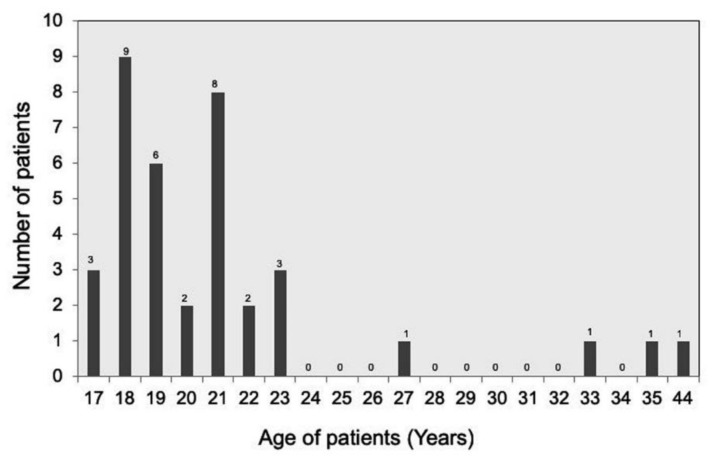
Age distribution of the 37 patients at the time of implant insertion.

Overall, four patients had syndromes associated with hypodontia. To assess possible genetic factors related to the presence of hypodontia, patients were asked about family history of hypodontia (parents, grandparents, siblings, and siblings of the parents). A total of 24 patients (64.9%) with hypodontia had a positive family history in this study; the remaining 13 patients had no family member with hypodontia. The age of the 37 patients at the time of implant placement ranged from 17 to 44 years, with a mean ± SD of 21.4 ± 5.6 and a median of 20 (Figure 3). Predominantly young patients (17–23 years, *n* = 33) received dental implants after the completion of cranial bone growth.

Patients with hypodontia either have class I, II, or III malocclusions. In this study, 30 patients reported with class I, one with class II, and six with class III malocclusion. Surgical management was necessary in seven patients who had class II and III, including bimaxillary advancement (four patients) and Le Fort 1 osteotomy (two patients), whereas bisagittal split osteotomy of the mandible was performed in one patient. Thirty-two patients received orthodontic treatment prior to implant placement (Table 3).

In 18 patients, alveolar ridge augmentation was performed either from the retro molar region (five patients (13.5%) and eight implants (5.2%)) or from the iliac crest (13 patients (35.1%) and 89 implants (57.4%)). Three different implant systems were used: 40 implants (25.8%) with Bego^®^ Semados (28 implants with Semados RI and 12 with Semados Mini/Bego Implant Systems, Bremen, Germany), 10 implants (6.5%) with Straumann Standard^®^ (Straumann AG, Basel, Switzerland), and 105 implants (67.7%) with Xive Plus^®^ (Dentsply Friadent, Mannheim, Germany). The time between implantation and follow-up was 5–189 months. The difference between follow-up examinations at the time of implantation constituted the in situ time of the implants (*n* = 155; mean age = 9.16 years, SD = 4.5 years, and median = 10.25 years).

Two implants were removed (1.3%): one after 6 months and one after 34 months due to periimplantitis. This resulted in survival rates of 97.7% and 100% for the upper and lower jaw, respectively. Survival rates for each implant company were as follows: Bego^®^, 97.5% (Bego Implant, Bremen, Germany); Xive^®^, 99% (Dentsply Friadent, Mannheim, Germany); and Straumann^®^, 100% (Straumann AG, Basel, Switzerland).

### 3.1. Patient-Related Parameters

**Figure 4:**
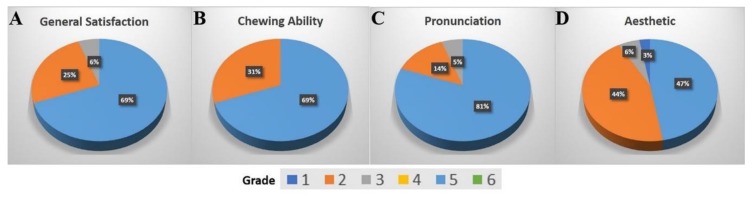
Satisfaction parameters of patients at the follow-up time graded using the German school grading system, in which 1 is the best and 6 is the worst. Left to right: (**A**) general satisfaction, (**B**) chewing ability, (**C**) pronunciation, and (**D**) aesthetics of patient (*n* = 36).

Functional and aesthetic outcomes were evaluated using a questionnaire. Of 37 patients, one could not answer the questionnaire because the dental implant was removed before the study. Figure 4 summarizes the patients’ answers using pie graphs in the categories of general satisfaction, chewing ability, pronunciation, and aesthetic outcome (*n* = 36) in percentages using the German School grading system.

Most of the patients (*n* = 25, 69.4%) reported general satisfaction with the dental implant, assigning an excellent grade; nine (25%) patients rated it good; and two (5.6%) patients rated it as acceptable. The chewing ability was rated excellent by 25 patients (69.4%) and good by 11 (30.6%). The pronunciation quality was rated excellent by 29 patients (80.6%), good by five patients (13.8%), and two (5.6%) rated it acceptable. In the aesthetic category, 17 (47.2%) patients rated the outcome excellent, 16 (44.4%) patients rated it good, and two (5.6%) rated it acceptable. Only one patient (2.8%) did not like the aesthetic outcome. In this patient, the implant surface was present under the mucosa. Regarding an improvement in quality of life due to the use of implants, patients provided very good and medium ratings (29 patients very good and eight medium).

Single-tooth crowns were used for 100 implants. In one patient with 15 implants, a double crown prosthesis was used for prosthetic reconstruction. Prosthetic restoration for 37 implants was an implant-supported bridge. Prosthetic complications were documented: crown loosening was observed in four implants, abutment loosening in one implant, and a screw fracture in one implant. All problems were reversible and could be repaired.

### 3.2. Analysis of Implant Success Using Albrektsson Criteria

**Figure 5:**
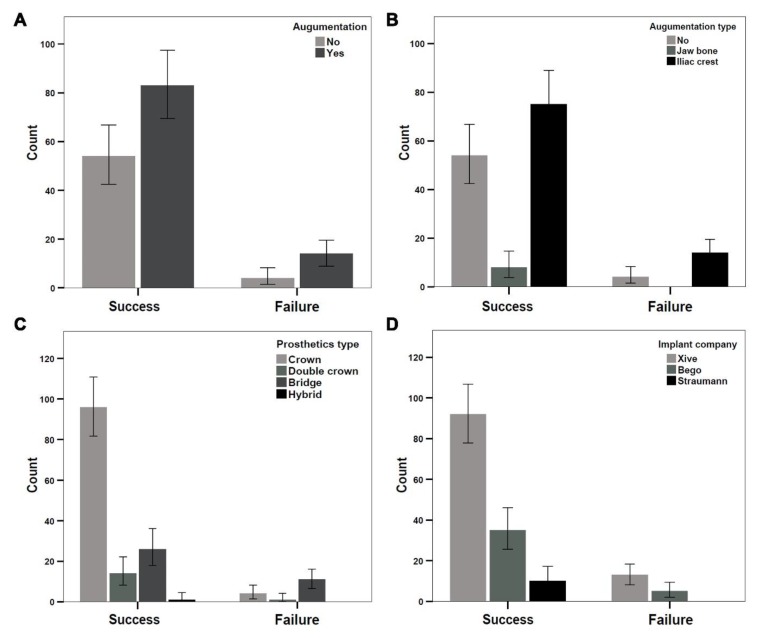
Frequency analysis revealed differences in the success and failure of implant materials based on the Albrektsson criteria. Implant success among patients was investigated through (**A**) the use of augmentation, (**B**) the choice of augmentation material, (**C**) the use of prosthetics, and (**D**) the choice of implant material.

The overall success of implants (*n* = 155) among patients (*n* = 37) was evaluated using Albrektsson criteria [31]. We evaluated 21 women and 16 men. Only two of 61 implants (3.27%) in women and 16 of 94 implants (17%) in men failed to meet the Albrektsson criteria. Frequency analysis was performed, and the results are presented as bar graphs with the standard error of the mean (Figure 5).

Bone augmentation was performed for 97 of 155 implant sites prior to implant placement. According to Albrektsson criteria, 14 implants failed in patients with augmented bone defects, and four implants failed in patients without bone augmentation (Figure 5A). Bone was harvested either from the jaw bone (eight implant sites) or iliac crest (89 implants sites); the use of the jaw bone as a grafting material showed no implant failure (Figure 5B), whereas implant failure was exclusively observed in areas grafted with iliac crest (14 of 89 = 15.7%; Figure 5B).

Four different prosthetic designs were used to treat the patients: crown, double crown, bridge, and hybrid. A dental crown was applied in 100 implants, of which three showed failure (Figure 5C). Bridge prosthetics were applied in 37 implants, of which 11 showed failure. Double crown prosthetics were applied in 15 implants, of which two implants showed failure (Figure 5C).

We analyzed four different implant systems in the patients: one system from Xive (Dentsply Friadent, Mannheim, Germany), two systems from Bego (Bego Implant, Bremen, Germany), and one system from Straumann (Straumann AG, Basel, Switzerland). Most patients (*n* = 22) were treated using Xive implants (*n* = 105); 10 of these implants showed failure (Figure 5D). A total of 13 patients were treated with 40 Bego implants, whereas two patients were treated with 10 Straumann implants. Implant failure was observed in five Bego implants, whereas no implant failures were observed in Straumann implants (Figure 5D).

**Figure 6:**
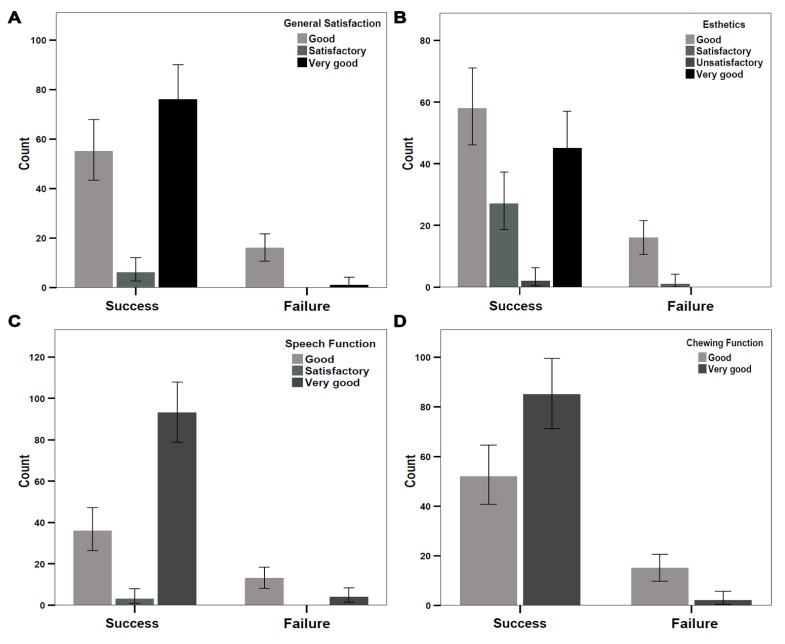
Frequency analysis revealed the implant success level in patients after rehabilitation. Implant performance and success was evaluated based on (**A**) the general satisfaction of patients, (**B**) the grading of aesthetics, (**C**) speech function, and (**D**) chewing ability after implantation.

Individual implant performance was evaluated among patients based on general satisfaction (with or without pain), aesthetics, speech, and chewing function (Figure 6). General satisfaction levels among patients were graded as satisfactory, good, and very good; 77 and 71 implants were graded as good and very good, respectively, whereas six implants were graded as satisfactory (Figure 6A). Of the 72 implants, 17 that were graded as very good showed implant failure according to the Albrektsson score. Only one implant failure was observed in implants graded as good; no implant failures were observed in the implants graded as satisfactory. The reliability of new implants was further tested by comparing aesthetic outcomes reported by patients (Figure 6B); these outcomes were categorized as unsatisfactory, satisfactory, good, and very good. Two implants were evaluated by the patients as unsatisfactory in their aesthetics, neither of which failed according to Albrektsson criteria. A total of 27 patients reported satisfactory aesthetics with one failure in implant success (Figure 6B). We found that 74 implants were graded as having good aesthetics and 47 implants were graded as having very good aesthetics. Of the 74 implants with good aesthetics, 16 implants showed failure. However, no implant failures were observed in the implants with very good aesthetics (Figure 6B). Speech and chewing functions were assessed among patients with implants. Speech function was graded as satisfactory, good, and very good. Of the implants, 97 were graded as very good, and no implant failures were observed (Figure 6C); 49 implants were graded as good, and 13 of these showed implant failure; the remaining three implants showed satisfactory speech function with no implant failures (Figure 6C). Chewing function was graded as good and very good (Figure 6D). Of the implants, 87 were graded as very good and 67 implants were graded as good; of the 87 implants, only two showed failure per the Albrektsson criteria, whereas 15 of the 67 implants showed failure (Figure 6D).

## 4. Discussion

The aim of treatment of hypodontia patients by replacing missing teeth is to restore functions such as chewing and speech. However, patients strongly value the aesthetic restoration of missing teeth. Aesthetics in the anterior region are invaluable for patient satisfaction. Unfortunately, conventional prosthetics solutions often yield unsatisfactory results and depend on preparing healthy teeth next to those that are missing. Therefore, patients with a large pulp chamber might need further treatment [19]. Although the use of adhesive bridges might avoid the preparation of healthy teeth [5], this is usually limited to managing a single missing tooth [32]. However, to lessen the bone loss that can result from bridge restoration, dental implantation is a useful alternative [33]. One advantage of dental implantation is the use of existing dental implants as an anchor for prosthetic choices in cases of further tooth loss next to the implant [5].

In this study, orthodontic treatment was performed in 32 patients (86%) before the implant placement. Early and proper orthodontic intervention can prevent bone loss in the hypodontic areas and create root parallelism to facilitate implant insertion surgery [16]. The most common orthodontic strategy for bone and keratinized mucosa preservation include orthodontic extrusion of primary teeth in infra-occlusion, delayed orthodontic space opening, orthodontic gap closure in risky implant regions, and the use of a rigid retainer to maintain the implant sites [16,34,35,36,37].

Bone augmentation surgeries in selected patients can be substituted using the orthodontic implant site switching (OISS) technique. The technique results in moving the tooth to the adjacent atrophic alveolar ridge to leave a gap with adequate bone, thereby leaving the original tooth position as a good quality region for successful implant placement and avoiding bone augmentation [35,38,39,40]. Although this is time-consuming and might result in the alteration of the periodontal support of the moved tooth, OISS can be used in hypodontia patients in the case of atrophic alveolar ridges. In such cases, donor-site morbidity of the autologous bone graft surgery can be avoided [35,40,41,42].

One of the limitations in this study is the missing information regarding orthodontic treatment. Many patients were referred to the university hospital from different orthodontists, and this led to difficulties in data collection.

Orthognathic surgeries to treat malocclusion were found in seven out of 37 patients (six class III and one class II). This result aligns with other studies that reported a high prevalence rate of hypodontia in patients with class III malocclusion [41]. 

Although difficult to interpret, the subjective assessment of patient satisfaction is important. In the context of implant success criteria, patient satisfaction is valuable [43]. To the best of our knowledge, only two studies regarding patient opinion of dental implants in cases of hypodontia have been published. Finnema et al. assessed patient satisfaction using a 10-point score (1 = very poor, 10 = excellent). The results showed that the patients were satisfied (seven to nine points) and more self-confident [44]. Zou et al. examined patient satisfaction, including aesthetics, denture comfort, and speech, using a three-point score (0 = unsatisfied, 1 = satisfied, and 2 = very satisfied); none of the patients were unsatisfied in that study [45]. However, neither study correlated the subjective satisfaction score with a well-known and dependable score of implant success. In the present study, implants were evaluated in terms of success according to Albrektsson criteria [31]. The subjective satisfaction of patients was obtained using questionnaires based on the literature [22,46,47,48,49]. Success criteria were then corroborated with patient satisfaction. Chewing function, speech ability, and aesthetics were assessed. The evaluation was based on the German school grade system (grade 1 to 6). In these categories, no grade was worse than satisfactory, except in a patient who was dissatisfied with aesthetics due to the visibility of implant material through the mucosa. In general, patient satisfaction in this study was high and comparable to that reported in similar studies [44,45].

Recently, we showed that dental implant survival did not necessarily constitute success, although only when Albrektsson criteria are applied to the investigated dental implants [30]. The augmentation procedure used to insert the implants (*n* = 97) was either from the retromolar region (*n* = 8) or from the iliac crest (*n* = 89). The area used for harvesting bone was selected based on the severity and size of bony defects. In case of a small and medium bone atrophy with two or fewer missing teeth, autografts were taken from the angle of the mandible [28]. However, bone from the iliac crest was used for augmentation if more than two teeth were missing and/or a combination of vertical and horizontal atrophy was diagnosed [50]. However, hypodontia commonly exhibits multiple missing teeth and complex atrophy, resulting in a higher number of implants inserted in iliac crest-augmented jaw bone. This may also explain why no unsuccessful implants were found in augmented jaws from the retromolar region. Each augmentation procedure involved different bone characteristics. Whereas iliac crest bone has more cancellous bone with higher bone resorption potential [51,52], compact bone (e.g., mandible and tabula externa) has slower resorption rates, which is preferable for long-term implant success [53].

One objective of this study was to correlate the type of prosthetics with implant success. Of 18 unsuccessful implants, 11 were bridges (61%), three were crowns (16.6%), and two were double crowns (11%). Altogether, 100 crowns, 37 bridges, and 15 telescopic crowns were designed for all patients. The pronounced failure incidence in the bridge design may be an indication of the difficulty in the maintenance of good oral hygiene [54].

Although patient satisfaction is important for assessment of procedural outcome [30], purely objective assessments are generally used as success criteria. We compared patient satisfaction with Albrektsson criteria for implant success. Of 18 unsuccessful implants, 17 of the patients reported very good satisfaction with the implant. Similar results were found in the subjective aesthetics component from the patient perspective, where 16 failed implants received good aesthetic evaluations. Speech and chewing functions showed similar results with 13 and 15 implant failures, respectively; all were rated as good by the patients. These results suggest that the objective success criteria used by professional dental implantologists may differ from the subjective consideration of patients. Therefore, a success scale is needed for dental implants that combine both aspects.

## 5. Conclusions

Despite unfavorable conditions with limited bone availability for the placement of dental implants in patients with hypodontia, we found a very good implant survival and success rate. Patients’ general satisfaction rates were high. All patients could eat a normal diet with good chewing ability. Pronunciation ability and aesthetics were acceptable for all patients. Dental implants constitute a standard therapy with high clinical, functional, and aesthetic treatment outcomes in patients with hypodontia. The current study suggests the need for a new assessment system of dental implants, which corroborates clinical parameters with patient contentment.

## Figures and Tables

**Table 1:** Overview of patients regarding number, sex, drop-outs, and implant removal.

	Number of Patients	Number of Implants
Total number of patients	43	165
Men	18	97
Women	25	68
Drop-outs	6	10
Investigated patients	37 *	155
Removed implants	2	2
Implant failure (Albrektsson)	7	18

* A total of 42 patients were selected with the inclusion criteria; however, only 37 patients who responded to the questionnaire and follow-up were investigated. Those six patients were considered drop-outs; therefore, they were not included in the statistical analysis. The removed implants were considered only in the investigated patients.

**Table 2:** Patients’ medical condition, allergies, and smoking behavior recorded prior to treatment.

General Disorders, Allergies, and Smoking Behavior	No. Patients
Cleft lip/palate	2
Diabetes Type 2	2
Bronchial asthma	1
Blood clotting disorder	1
Ectodermal dysplasia	2
Hypothyroidism	3
Allergy: penicillin	1
Smoking	10

**Table 3:** The relationship between the aesthetic treatment outcomes and the orthodontic treatment prior to implant placement.

No. of Patients (%)	Aesthetics	Orthodontic Treatment Prior to Implant Placement?	
		No	Yes
17 (47.22%)	Very good	2 (5.55%)	15 (41.66%)
16 (44.44%)	Good	2 (5.55%)	14 (38.88%)
2 (5.55%)	Satisfactory	1 (2.77%)	1 (2.77%)
1 (2.77%)	Unsatisfactory	0	1 (2.77%)

## Supplementary Materials

The following are available online at https://www.mdpi.com/2077-0383/8/10/1687/s1, Table S1: Number of implants per region and type of replaced tooth.
