# Supplementary material for: Oral Rehabilitation of Hypodontia Patients Using an Endosseous Dental Implant: Functional and Aesthetic Results
Source: J Clin Med. 2019 Oct 15;8(10):1687. doi: 10.3390/jcm8101687 (PMC6832447; doi:10.3390/jcm8101687)
Supplement: Supplementary file 1 [file jcm-08-01687-s001.pdf]

Journal of
Clinical Medicine

MDPI

Table S1. Number of implants per region and type of replaced tooth.

Type of teeth	Position	Number	Total
Incisors	12 (right maxillary lateral incisor)	17	50
	22 (left maxillary lateral incisor)	17	
	32 (left mandibular lateral incisor)	6	
	31 (left mandibular central incisor)	3	
	41 (right mandibular central incisor)	2	
	42 (right mandibular lateral incisor)	5	
Canines	13 (right maxillary canine)	6	21
	23 (left maxillary canine)	6	
	33 (left mandibular canine)	5	
	43 (right mandibular canine)	4	
Premolars	15 (right maxillary second premolar)	10	68
	14 (right maxillary first premolar)	7	
	24 (left maxillary first premolar)	6	
	25 (left maxillary second premolar)	10	
	35 (left mandibular second premolar)	14	
	34 (left mandibular first premolar)	5	
	44 (right mandibular first premolar)	4	
	45 (right mandibular second premolar)	12	
Molars	17 (right maxillary second molar)	3	16
	16 (right maxillary first molar)	2	
	26 (left maxillary first molar)	1	
	27 (left maxillary second molar)	1	
	37 (left mandibular second molar)	2	
	36 (left mandibula first molar)	3	
	46 (right mandibular first molar)	3	
	47 (right mandibular second molar)	1	
